# Supplementary material for: Multiplexed Detection of Pancreatic Cancer by Combining a Nanoparticle-Enabled Blood Test and Plasma Levels of Acute-Phase Proteins
Source: Cancers (Basel). 2022 Sep 25;14(19):4658. doi: 10.3390/cancers14194658 (PMC9563576; doi:10.3390/cancers14194658)
Supplement: Supplementary file 1 [file cancers-14-04658-s001.zip › cancers-1837328-supplementary.pdf]

Supplementary Material

Multiplexed detection of pancreatic cancer by combining nanoparticle-enabled blood test and plasma levels of acute-phase proteins

Damiano Caputo [1,2], Alessandro Coppola [2], Erica Quagliarini [3], Riccardo Di Santo [3], Anna Laura Capriotti [4], Roberto Cammarata [2], Aldo Laganà [4], Massimiliano Papi [5,6], Luca Digiacomo [3], Roberto Coppola [1,2], Daniela Pozzi [3,*] and Giulio Caracciolo [3,*]

[1] University Campus Bio-Medico di Roma, Via Alvaro del Portillo 200, 00128 Rome, Italy
[2] General Surgery, Fondazione Policlinico Universitario Campus Bio-Medico, Via Alvaro del Portillo 200, 00128 Rome, Italy
[3] NanoDelivery Lab, Department of Molecular Medicine, Sapienza University of Rome, Viale Regina Elena 291, 00161 Rome, Italy
[4] Department of Chemistry, Sapienza University of Rome, P.le A. Moro 5, 00185 Rome, Italy
[5] Dipartimento di Neuroscienze, Università Cattolica del Sacro Cuore, Largo Francesco Vito 1, 00168 Rome, Italy
[6] Fondazione Policlinico Universitario A. Gemelli IRCSS, 00168 Rome, Italy
[*] Correspondence: daniela.pozzi@uniroma1.it (D.P.); giulio.caracciolo@uniroma1.it (G.C.).

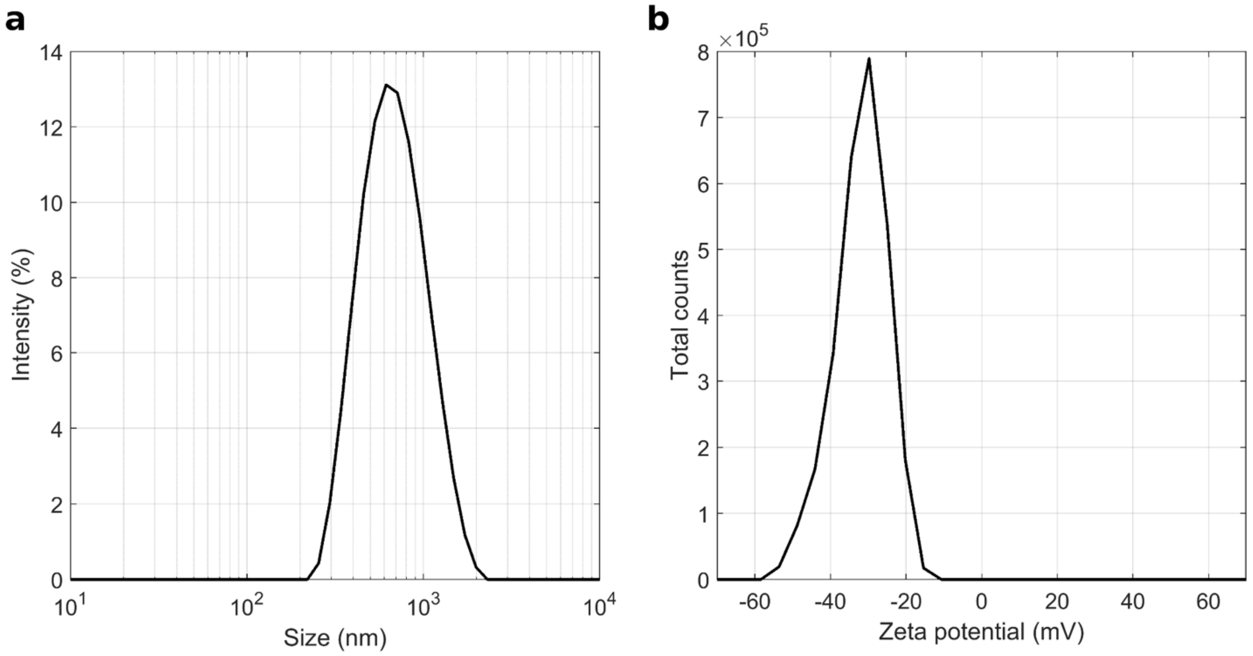

Figure S1. Size (a) and zeta-potential (b) distributions of the GO nanosheets used in this study.

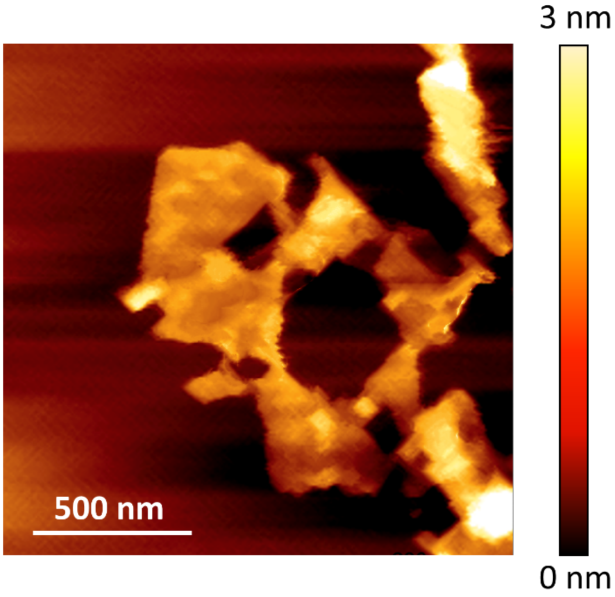

Figure S2. Representative AFM image of GO nanoflakes.

Table S1. Size, polydispersity index (PdI) and zeta potential of the graphene oxide nanosheets used in the study. Mean values were obtained as the average over three independent measurements.

	Mean	Standard deviation
Size (nm)	667	9
PdI	0,294	0,031
Zeta potential (mV)	-32,4	1,7

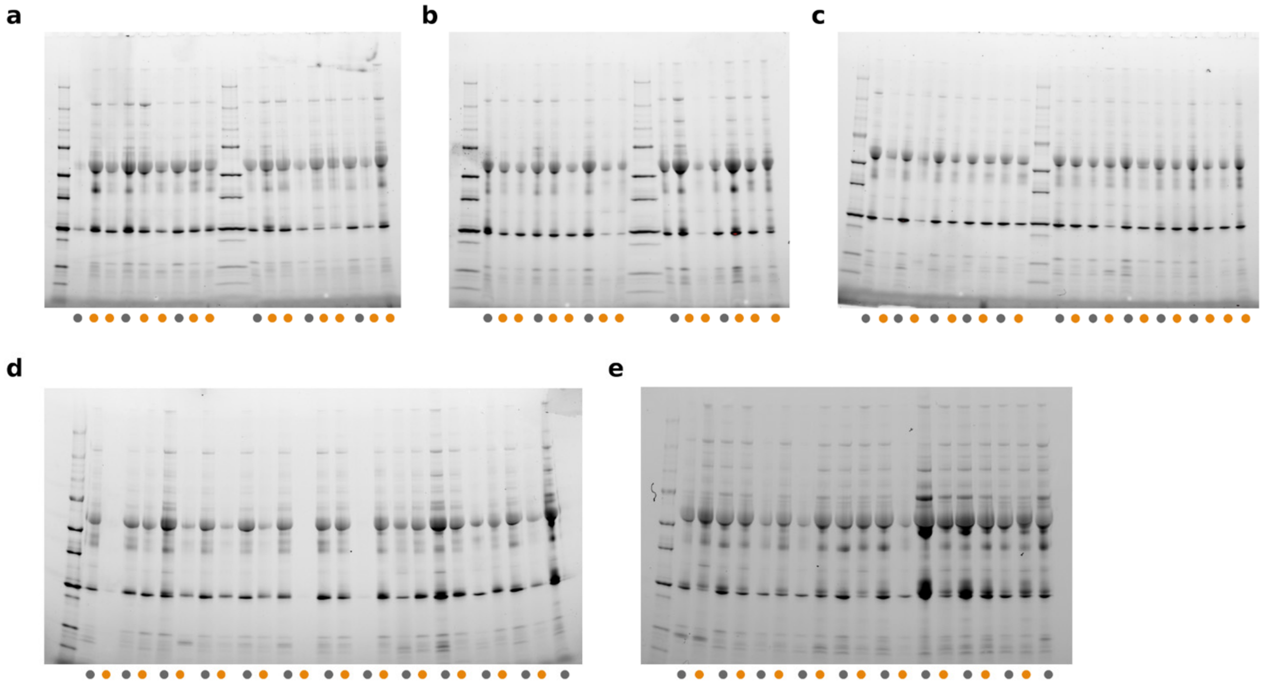

Figure S3. One-dimensional (1D) SDS-PAGE gel images. In the gels each lane refers to a single human subject, being either a PDAC patient (orange points) or a healthy volunteer (black points). Not labelled lanes are the protein ladders.

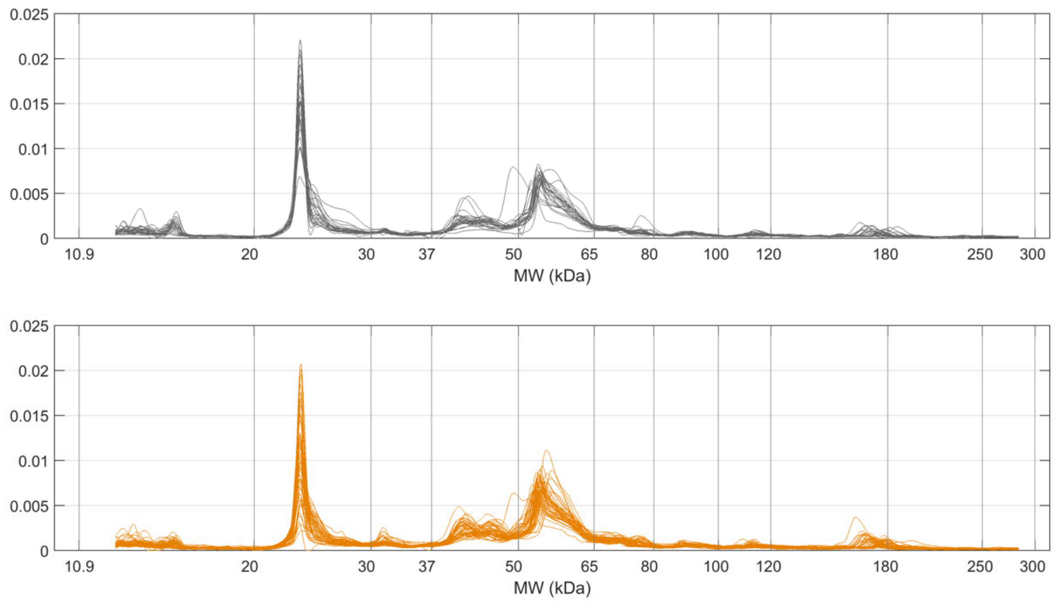

Figure S4. One-dimensional (1D) profiles obtained by densitometric analysis of the 1D SDS-PAGE gel images shown in Figure S3.

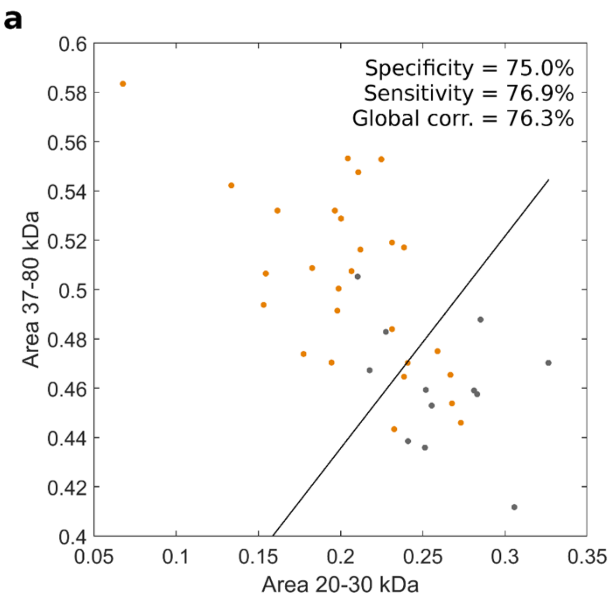

Figure S5. Sex-disaggregated scatter plots of the integral areas showing the largest difference between the 1D SDS-PAGE profiles for women (panel a) and men (panel b). In both the panels the solid black lines describe the result of the linear discriminant analysis for the distributions. No relevant differences between samples of different sex were found.
